# Concurrent Liver Hodgkin Lymphoma and Nodular Regenerative Hyperplasia on an Explanted Liver with Clinical Diagnosis of Alcoholic Cirrhosis at University Hospital Fundación Santa Fe de Bogotá

**DOI:** 10.1155/2014/193802

**Published:** 2014-01-05

**Authors:** R. López, L. Barrera, A. Vera, R. Andrade

**Affiliations:** ^1^Pathology and Clinical Laboratory Department, University Hospital Fundación Santa Fe de Bogotá, Calle 119 No. 7–75, Bogotá 110111, Colombia; ^2^Transplant Service, University Hospital Fundación Santa Fe de Bogotá, Calle 119 No. 7–75, Bogotá 110111, Colombia; ^3^School of Medicine, Universidad de los Andes, Cra 1 No. 18A-12, Bogotá 111711, Colombia

## Abstract

Liver involvement by Hodgkin lymphoma (HL) is well documented. However, secondary liver failure to this neoplastic process is rare and usually presents late in the course of the disease. We present a case of a HL associated with nodular regenerative hyperplasia (NRH) diagnosed on an explanted liver from a 53-year-old patient with clinical diagnosis of alcoholic cirrhosis. Hematoxylin and eosin stain (H&E) showed abnormal liver architecture with hepatocytes nodules highlighted by reticulin stain with absent fibrosis on the trichrome stain. The portal spaces had diffuse infiltration by Reed-Sternberg cells positive for CD15, CD30, and latent membrane protein (LMP) on immunohistochemical studies. The patient also had a concurrent hilar lymph node biopsy that also showed HL involvement. Liver failure as the initial presentation of Hodgkin' lymphoma is rare. We believe that more research about the utility of performing liver biopsies in patients candidates for transplantation with noncirrhotic hepatic failure is needed in order to establish the etiology and the optimal treatment.

## 1. Introduction 

Liver involvement by Hodgkin's lymphoma (HL) is well documented [1] and secondary liver failure usually presents late in the course of the disease; however, there are some HL cases that present as a primary acute liver failure [2]. It is believed that hepatic failure by HL is due to bile duct destruction and direct tissue infiltration by the neoplastic cells [1, 2].

## 2. Case Report

The clinical history is that of a 53-year-old man who presented to our hospital with hepatic encephalopathy; his clinical history was positive for 18 years of alcohol abuse. He referred; recurrent ascites and six episodes of upper gastrointestinal bleeding from portal origin. Laboratory tests showed aspartate aminotransferase (AST) 35 U/L (5–34 U/L), alanine transaminase (ALT) 31 U/L (<55 U/L), alkaline phosphatase 215 U/L (woman >15 years old: 40–150), gamma-glutamyltransferase (GGTP) 368 U/L (Adult woman: 9–36), serum bilirubin 1.6 mg/dL (Total 0,2–1 mg/dL), direct bilirubin 0.76 mg/dL (0.10–0.5 mg/dL), indirect bilirubin 0.85 mg/dL, albumin 3.5 g/dL (3.4–5.4 g/dL), platelets 147 (10)^3^/mm^3^ (150–450 (10)^3^/mm^3^), creatinine 0.71 mg/dL (0.7–1.3 mg/dL), prothrombin time (PT) 14,4 sec (Control 11.1), partial thromboplastin time (PTT) 33.2 sec (Control 28,1), and INR 1.29. The patient was on propranolol 40 mgr once a day, gabapentin 600 mgr three times a day, and omeprazole 20 mgr once a day. Abdominal computed tomography (CT) showed diffuse hypervascular lesions on the liver parenchyma, portal hypertension, splenomegaly, ascites, esophageal, and splenic varices.

Clinical diagnosis based on clinical history, physical exam, and liver findings of alcoholic cirrhosis included the patient in liver transplant list. An orthotopic liver transplant was performed without complications; however, 1 week after transplantation a HL and nodular regenerative hyperplasia (NRH) were diagnosed on the explanted liver. Extension studies confirmed a stage IV-B mixed cellularity HL (hepatic, hilar ganglion and bone compromise (vertebral column)); Bone Marrow Aspiration was reported as negative for lymphoproliferative process. First three cycles of adriamycin, blemomycin, vinblastine, dacarbazine (ABVD) were administered without significant complication. Four months later during fourth cycle, patient presented hyperbilirubinemia and treatment was suspended, he required biliar pathway expansion thought percutaneous drainage. A close followup was done during the next 5 months when he was hospitalized to perform a new bone marrow biopsy and myelogram, both without evidence of active lymphoma. Unfortunately contact with the patient and family was lost after almost 10 months of transplantation.

## 3. Materials and Methods

**Figure 1:**
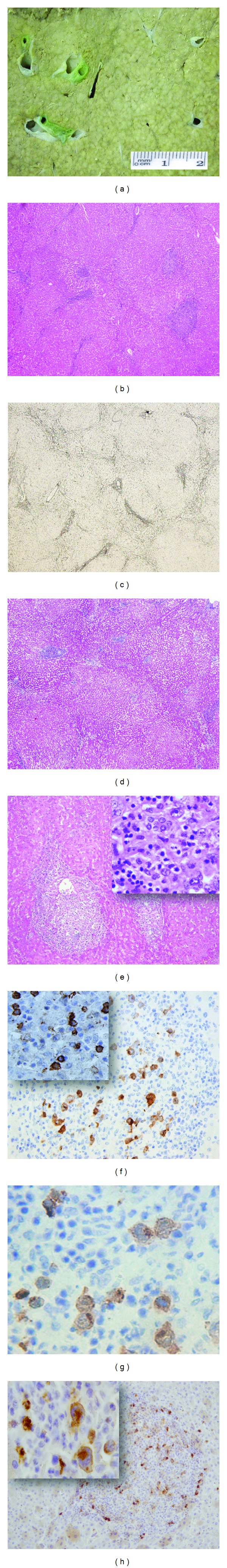
(a) Cut surface of the liver with a micronodular appearance. (b) H&E 4X, Highlighting nodularity. (c) Reticulin stain 4X, again highlighting nodularity. (d) Trichrome satin 4X, showing absent fibrosis. (e) H&E 4X, portal space with chronic inflammatory infiltrate, inset 40X, Reed-Stenberg cells on a lymphoid background. Immunohistochemical studies: (f) LMP 40X, inset CD 20 100X. (g) CD 30 100X, presenting Positive Reed Stenberg cells and (h) CD 15 40X and Inset CD 15 100X, presenting Positive Reed Stenberg cells.

Grossly a 1438 gr explanted liver that measured 20 × 14 × 8 cm was received. The surface had a brown to purple color with nodular appearance. Cut surface showed a micronodular liver parenchyma, with nodules ranging from 0.1 to 0.3 cm and no dominant lesions were noted; representative sections were submitted (Figure 1(a)).

## 4. Microscopic Examination 

H&E slides showed an abnormal liver architecture given by a slight nodularity (Figure 1(b)). The hepatocytes within the nodules were enlarged with hypertrophic nuclei, and the hepatocytes between the nodules looked small and atrophic. Reticulin stain highlighted the nodular parenchyma (Figure 1(c)) with no evidence of fibrosis on trichrome stain (Figure 1(d)). These findings were compatible with NRH. Also the portal spaces had diffuse and prominent infiltration by atypical large cells morphologically consistent with Reed-Sternberg cells in a lymphoid background (Figure 1(e)). Immunohistochemical studies showed that neoplastic cells were positive for LMP (Figure 1(f)), CD 20 (Figure 1(f) inset), CD 30 (Figure 1(g)), and CD 15 (Figure 1(h)). After HL diagnosis a hilar lymph node showed compromise by HL, mixed cellularity subtype.

## 5. Discussion

NRH is an increasingly recognized cause of noncirrhotic portal hypertension and was first described on 14% of liver biopsies from patients with portal hypertension without cirrhosis [3]. It is believed that this disease is under-diagnosed; as a matter of fact a study by Wanless found NRH in 64 patients (2.6%) of a total of 2500 autopsies [4]. Interestingly enough almost all the cases were associated with systemic disorders. The same study reported that NRH prevalence was 7 times higher in patients older than 80 years when compared with patients who were under 60 years of age. It is believed that NRH is secondary to chronic hepatic blood flow alterations [5, 6], probably due to portal vein damage [7].

Several diseases including some rheumatological and hematological, drugs and portal vein congenital abnormalities have been associated with NRH [5]. Among the associated hematological disorders is been described concurrence with myeloproliferative diseases including polycythemia vera, chronic myelogenous leukemia, essential thrombocythemia, and HL [3, 8–10].

Association between NRH and HL is usually seen in advanced HL stages [1, 9], however liver failure as the initial presentation of HL is rare and accounts for less than 4% of patients [1] as seen in our case. In a review of the literature acute liver failure secondary to HL was due either to vanishing bile duct syndrome or to direct neoplastic cellular proliferation in the liver parenchyma and portal spaces. Hemolysis, infectious hepatitis, toxicity from chemotherapy, and periportal lymph node involvement with compression to the extrahepatic biliary system are other manifestations of HL liver involvement [11].

Regarding vanishing bile duct syndrome, even though the mechanism of injury to the liver is not well understood, it seems to be related to an inflammatory reaction within the bile ducts (in the absence of lymphoma's cells infiltration) manifested as an acute cholestatic hepatitis [9], which can progress to bile duct destruction. Also, the neoplastic cell infiltrate has shown hepatocyte necrosis/regeneration, central venulitis and cholangiolar proliferation [2].

We found only one case report from The University Hospital of Andalucía (Madrid, Spain): a 63-year-old patient who presented with portal hypertension, progressive liver failure and death, in whom a postmortem liver biopsy (LB) showed features of HL [12]. The histological findings revealed that the mechanisms producing the portal hypertension were directly related to portal infiltration/obliteration of the hepatic tissue.

Indications for biopsy in liver dysfunction patients who are candidates for transplantation are currently controversial. According to literature in the context of alcoholiccirrhosis, diagnosis can be made through clinical observations with high accuracy [13]. In our hospital experience over more than 23 years of liver transplant nearly 90% of transplant candidates do not undergo LB as a part of the transplant evaluation for two main reasons: First, clinically the patients are at risk of bleeding, embolic disorders, arterioportal fistula and infections [14] and second, the histopathological information provided by a LB in advanced cirrhosis has limited utility and in general would not change the treatment course.

## 6. Conclusion

NRH is a well-recognized cause of noncirrhotic portal hypertension, mimics alcoholic cirrhosis and should be included in the differential diagnosis. Advanced stages of HL have been related to hepatic NRH [1, 9]. However we believe that more research about the utility of performing liver biopsies in patients with noncirrhotic hepatic failure is needed. Currently proper clinical information is decisive in determining the etiology and possible treatment options.
